# Phylogenetic and Molecular Clock Analysis of Dengue Serotype 1 and 3 from New Delhi, India

**DOI:** 10.1371/journal.pone.0141628

**Published:** 2015-11-04

**Authors:** Nazia Afreen, Irshad H. Naqvi, Shobha Broor, Anwar Ahmed, Shama Parveen

**Affiliations:** 1 Centre for Interdisciplinary Research in Basic Sciences, Jamia Millia Islamia, New Delhi, India; 2 Dr. M.A. Ansari Health Centre, Jamia Millia Islamia, New Delhi, India; 3 Department of Microbiology, Faculty of Medicine and Health Science, Shree Guru Gobind Singh Tricentenary University, Gurgaon, Haryana, India; 4 Protein Research Chair, Department of Biochemistry, College of Science, King Saud University, Riyadh, Saudi Arabia; Emory University School of Medicine, UNITED STATES

## Abstract

Dengue fever is the most prevalent arboviral disease in the tropical and sub-tropical regions of the world. The present report describes molecular detection and serotyping of dengue viruses in acute phase blood samples collected from New Delhi, India. Phylogenetic and molecular clock analysis of dengue virus serotype 1 and 3 strains were also investigated. Dengue virus infection was detected in 68.87% out of 604 samples tested by RT-PCR between 2011 & 2014. Dengue serotype 1 was detected in 25.48% samples, dengue serotype 2 in 79.56% samples and dengue serotype 3 in 11.29% samples. Dengue serotype 4 was not detected. Co-infection by more than one dengue serotype was detected in 18.26% samples. Envelope gene of 29 DENV-1 and 14 DENV-3 strains were sequenced in the study. All the DENV-1 strains grouped with the American African genotype. All DENV-3 strains were found to belong to Genotype III. Nucleotide substitution rates of dengue 1 and 3 viruses were determined in the study. Time to the most recent common ancestor (TMRCA) of dengue 1 viruses was determined to be 132 years. TMRCA of DENV-3 viruses was estimated to be 149 years. Bayesian skyline plots were constructed for Indian DENV-1 and 3 strains which showed a decrease in population size since 2005 in case of DENV- 1 strains while no change was observed in recent years in case of DENV-3 strains. The study also revealed a change in the dominating serotype in Delhi, India in recent years. The study will be helpful in formulating control strategies for the outbreaks. In addition, it will also assist in tracking the movement and evolution of this emerging virus.

## Introduction

Dengue is a mosquito-borne acute viral infection prevalent in tropical and subtropical areas of the world. It is caused by an RNA virus belonging to the family Flaviviridae genus Flavivirus. Dengue virus (DENV) exists as four serotypes which are implicated in worsening of the disease in some cases through the mechanism of antibody dependent enhancement. As a result, secondary infection by a different serotype is often associated with severe dengue. Common symptoms of dengue include fever, rash, headache, nausea, vomiting and weakness.

Dengue infection is diagnosed by virus culture, RT-PCR, NS1 antigen ELISA and IgG and IgM ELISA. During the first 4–5 days of the disease onset, virus culture, RT-PCR and NS1 antigen ELISA are the methods of choice for diagnosis of dengue as the virus is detectable in plasma, serum, circulating blood cells and other tissues. After this duration serological methods are preferred as the host antibodies could be detected by this time [1]. Collection of acute and convalescent sera is recommended to demonstrate the appearance of virus-specific antibodies following infection [2].

In the present report, we have done detection and serotyping of dengue strains in clinical samples which were collected from New Delhi, India.Dengue serotypes 1, 2 and 3 have been detected in Delhi, India in the last 2 decades and have been characterized genetically[3–5]. Genotype American African of DENV-1 and Genotype III of DENV-3 have been detected in India [3,4]. The present study also reports genetic characterization of DENV-1 and 3 strains circulating in 2012–2014 in Delhi, India. We also infer nucleotide substitution rates and time to the most recent ancestor of the circulating lineages through Bayesian inferences. Past population dynamics of Indian DENV-1 and 3 strains based on Bayesian skyline plots were also investigated.

## Materials and Methods

### Sample collection

The study was approved by Institutional Ethics Committee, Jamia Millia Islamia and was done in accordance with the World Medical Association Declaration of Helsinki. Blood samples were collected from the symptomatic dengue patients attending Out Patient Department of Ansari Health Centre, Jamia Millia Islamia. Written informed consent in English or Hindi (local language) was obtained from the study subjects.

### RT-PCR for detection of Dengue viruses in clinical samples

Serum separation from blood samples was done by centrifuging at 3000 rpm for 10 minutes at 4°C. Serum samples were stored at -80°C until further use. RNA was extracted from serum samples using QIAamp Viral RNA Mini kit (Qiagen, Hilden, Germany) as per the manufacturer’s instructions. Dengue virus specific RNA was detected by semi nested RT-PCR method as reported by Lanciotti et al. [6] with some modifications [7,8]. External PCR was carried out using D1 &D2 primers which are specific to all 4 serotypes of the dengue virus. Amplicon of the external PCR was diluted in 1:5 ratio with autoclaved double distilled water. The diluted amplicon was used as template in the nested PCR. The D1 was used as forward primer and dengue serotype specific primers (TS1, TS2, TS3 and TS4) were used as reverse primers in this nested PCR. The nested PCR results in amplicons of different sizes specific to each serotype (485bp, 119bp, 290bp and 392bp for DENV 1–4 respectively). Amplicons were run on agarose gel and visualized with ethidium bromide in UV light.

### DNA sequencing of Dengue 1 and 3 viruses

E gene segments of 448bp and 582bp of dengue serotype 1 and 3 viruses respectively were amplified using another set of published primers for DNA sequencing [9,10]. Amplicons were run on 2% agarose gel. Specific bands were cut from the gel and amplicons were extracted using QIAquick Gel Extraction Kit (Qiagen, Germany) according to manufacturer’s instructions. Sequencing was done commercially (Xcelris Labs, Ahmedabad, India) in both forward and reverse directions.

### Phylogenetic analysis

Identity of the raw sequences was confirmed by running BLAST software available at NCBI website. Forward and reverse sequences were aligned and manually edited in GeneDoc (v2.7.000) software. The study sequences were aligned with other published sequences retrieved from GenBank using Clustal W [11] implemented in BioEdit (v7.0.9.0) [12]. The best fit substitution model was chosen by Akaike Information Criterion (AIC) using MODELTEST3.7 [13]. Maximum likelihood phylogenetic tree was constructed in Mega 6.06 software [14]. Bootstrapping with 1000 replicates was done to show support for each node in the tree.

### Bayesian MCMC analysis

Rate of nucleotide substitution and time to the most recent common ancestor (TMRCA) of DENV-1 and 3 strains were determined using Bayesian inferences implemented in BEASTv1.8.1 [15]. TN93 +G model was used as the nucleotide substitution model and among site rate variation model for Dengue 1 while GTR+G+I was selected for dengue 3 (selected by MODELTEST3.7). Both strict and relaxed (uncorrelated exponential and uncorrelated lognormal) molecular clocks [16] were used for the analysis. Constant size and Bayesian skyline coalescent tree priors were used in the study. The MCMC chain was run for 30,000,000 steps. The parameter values were sampled at every 3000 steps. The best fit model was chosen by Bayes factor [Log marginal likelihood (M1)-Log marginal likelihood(M2)]. Log marginal likelihoods were determined by stepping stone sampling. The model chosen as best for the data was performed in two separate runs and the resulting log files were combined using LogCombiner 1.8.1 (implemented in BEAST) with 10% burn-ins removed from each run. The resulting log files were analysed in the program Tracer 1.6 to ascertain convergence of the chain and to ensure that effective sample size of >200 for all parameters have been reached. The uncertainty in the parameter estimates were assessed by 95% HPD interval. The maximum clade credibility tree was generated by Tree Annotator 1.8.1 (available in BEAST), and the resulting tree file was visualized in the program FigTree 1.4.2. Support for the node on the tree was ascertained by the Bayesian posterior probability (BPP) values for each node. The BEAST package was also used to infer Bayesian skyline plots for Indian DENV- 1 and 3 strains. This analysis enabled a graphical depiction of changing levels of population size (Neτ, where Ne is the effective population size and τ the host-to-host generation time) through time.

## Results

### Dengue virus prevalence and serotypic distribution (2011–2014)

A total of 604 acute phase blood samples were collected during the four year prospective study carried out between 2011 & 2014. Information regarding age and duration of fever were available for 514 patients and regarding sex was available for 578 patients. The mean age of the study subjects was 22.52 ±13.41years. The male to female ratio was 1.69: 1. The duration of illness ranged from 0 to 15 days (mean ± SD; 3.48±2.38 days). Dengue virus infection was detected in 416 (68.87%) samples. Dengue serotype 1 was detected in 106 (25.48%) samples, dengue serotype 2 in 331 (79.56%) samples and dengue serotype 3 in 47 (11.29%) samples. Dengue serotype 4 was not detected in any of the samples. Seventy six (18.26%) samples showed co-infection by more than one dengue serotype. Dengue serotype 1 and 2 both were detected in 53 samples, dengue 1 and 3 in 3 samples and dengue 2 and 3 in 20 samples. Year wise results are shown in Table 1. Results obtained in the year 2011 and 2013 have been published previously [7,8]. Serotype distribution was detected to vary every year. Dengue serotype 1 was not detected in 2012 while serotype 3 was not detected in 2014. Dengue serotype 2 remained the predominant serotype in all the four years (Table 1).

**Table 1:** Year-wise results* of RT-PCR based detection and serotyping of Dengue viruses.

	2011	2012	2013	2014	Total
**Number of patients**	87	77	378	62	604
**Dengue Positive**	43 (49.42%)	45 (58.44%)	269 (71.16%)	59 (95.16%)	416 (68.87%)
**DENV-1**	18 (41.86%)	0	52 (19.33%)	36 (61.02%)	106 (25.48%)
**DENV-2**	21(48.83%)	29 (64.44%)	232 (86.25%)	49 (83.05%)	331(79.56%)
**DENV-3**	8 (18.60%)	16 (35.55%)	23 (8.55%)	0	47 (11.29%)
**DENV-4**	0	0	0	0	0
**Co-infection**	4 (9.30%)	9 (20.00%)	37 (13.75%)	26 (44.07%)	76 (18.26%)
**DENV-1 & 2**	1 (25%)	0	26 (70.27%)	26 (100%)	53 (69.74%)
**DENV-1 & 3**	2 (50%)	0	1 (2.70%)	0	3 (3.95%)
**DENV-2 & 3**	1 (25%)	9 (100%)	10 (27.03%)	0	20 (26.32%)

*including co-infections

### Phylogenetic analysis of DENV- 1 strains

**Fig 1:**
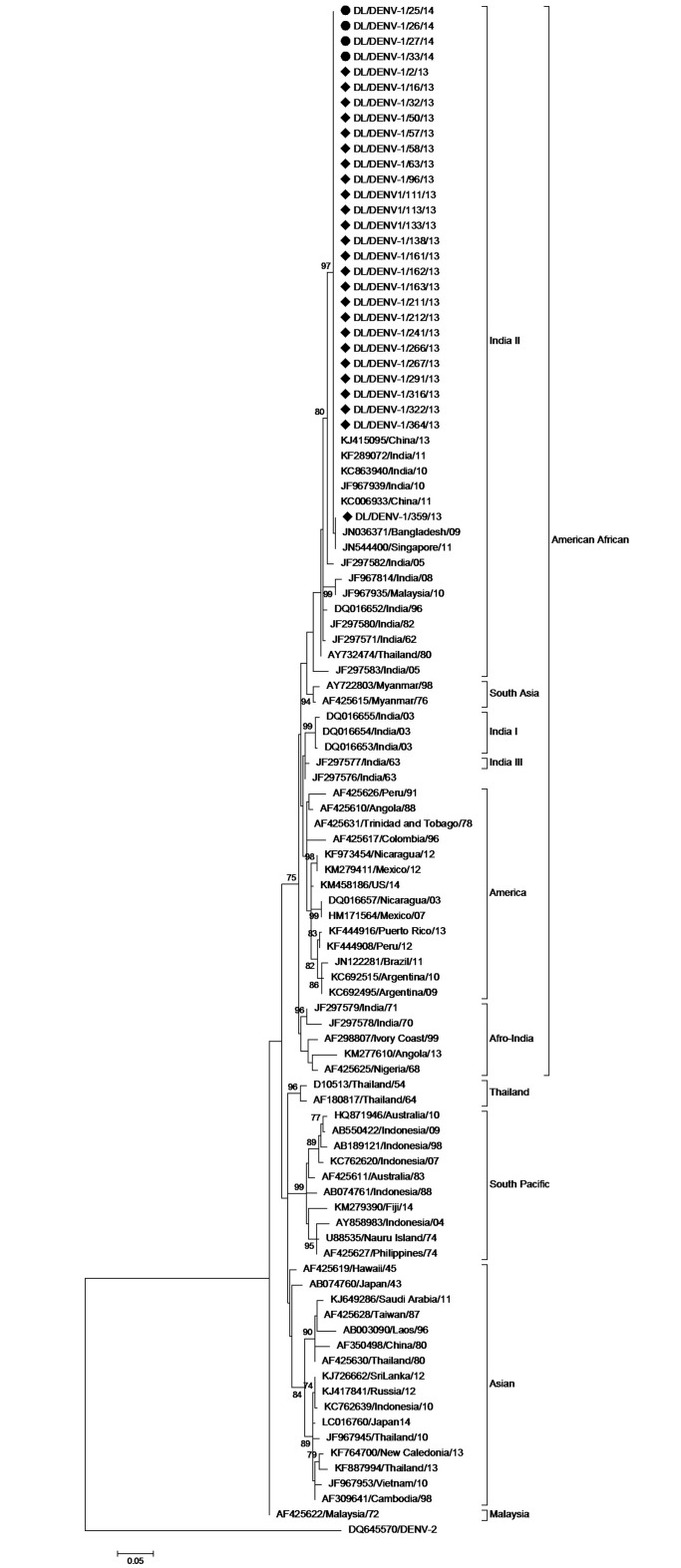
Maximum likelihood phylogenetic tree of DENV-1 strains. The strains sequenced in the study are marked by diamonds. Numbers on nodes indicate bootstrap support generated by 1000 replicates. Bootstrap values of >70 are shown.

E gene segments of 29 DENV-1 strains were sequenced in the present study. Twenty five strains were sequenced in 2013 while 4 strains were sequenced in 2014. The study sequences were deposited in the GenBank database with following accession numbers; GenBank: KJ729163-KJ729172 and KR091047- KR091065. Sequencing and phylogenetic analysis of the sequences with accession numbers KJ729163-KJ729172 have been described in our previous paper [7]. A 353 bp (117 amino acid) region of envelope gene corresponding to 1921–2273 bp of the DENV-1 genome and 332–448 amino acid of the E protein (numbering based on the prototype Nauru strain; GenBank Accession Number: U88535) was aligned. The study strains were found to group together with the American African Genotype (Fig 1) on phylogenetic analysis by Maximum Likelihood method. Patil et al. have described four lineages of Indian strains i.e. India I, II, III and Afro-India [17]. Strains of the present study clustered in Lineage India II of Patil et al. which is considered as an in situ evolving lineage. Six mutations i.e. PheE339Ile, SerE341Thr, AlaE371Thr, Val E382Ile, IleE438Val, Ile E441Val were detected in the study strains in comparison to the prototype strain. All these mutations were shown by earlier published sequences [18]. The study sequences showed nucleotide distance of up to 0.3% amongst themselves while amino acid sequences were identical. Strains from 2014 were identical to the predominant strain of 2013. The study strains were similar to recent strains reported from India, China, Bangladesh and Singapore (Fig 1).

### Bayesian MCMC analysis of DENV-1 strains

**Fig 2:**
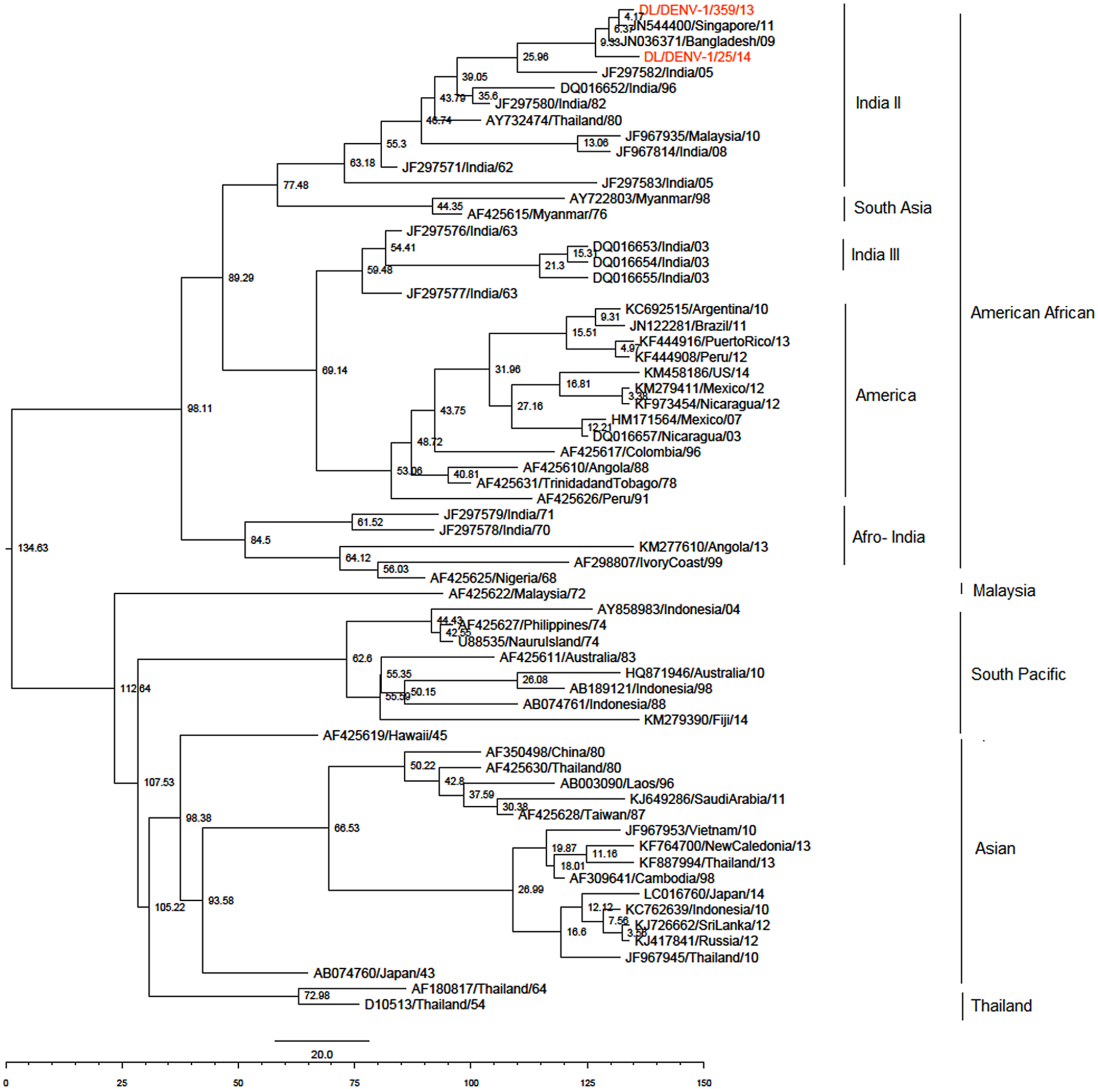
Maximum Clade Credibility tree of Dengue-1 viruses. Tree derived with the best fit model. Node ages are shown at each node.

**Fig 3:**
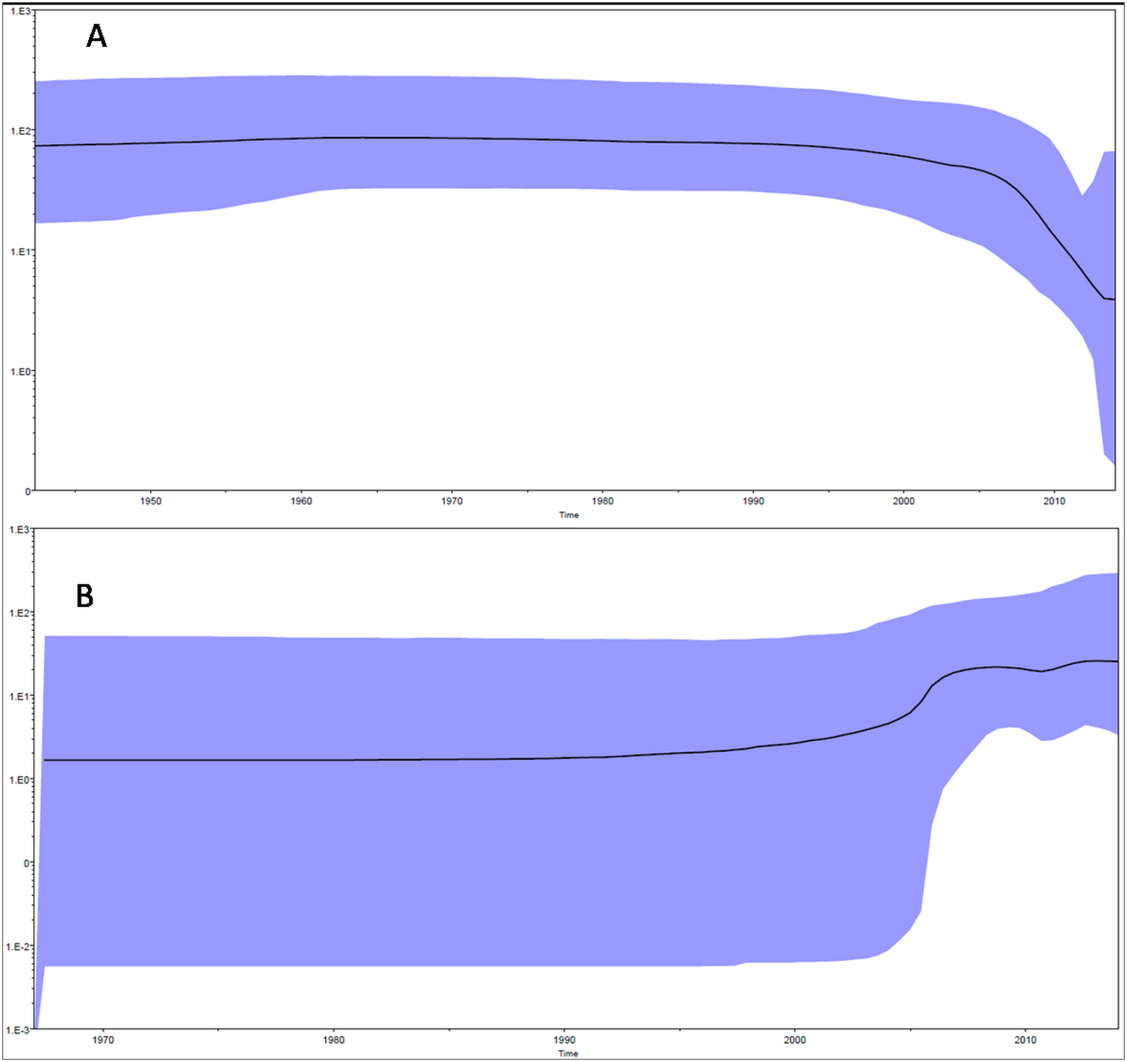
Bayesian skyline plots A) Indian DENV-1 strains B) Indian DENV-3 strains. X axis- time, Y axis-Neτ. Black solid line is the median estimate of Neτ. Blue shaded area shows 95% HPD.

Bayesian evolutionary analysis was conducted with the data used for maximum likelihood phylogenetic analysis. The estimated nucleotide substitution rate was lower than reported in literature (data not shown). So to correctly estimate the dates of divergene, we conducted the bayesian analysis with sequence alignment of nine study DENV-1 strains described in our previous publication [8]. Bayesian evolutionary analysis for DENV-1 was conducted using Tamura Nei 93 model of nucleotide substitution with gamma distributed rate variation among sites (4 categories) (TN93+G as selected by MODELTEST 3.7). Uncorrelated relaxed lognormal clock and Bayesian skyline tree prior was chosen as the best fit model as it was favoured by Bayes factor (Table 2). Maximum Clade Credibility Tree was constructed with this model as shown in Fig 2. The mean nucleotide substitution rate under the relaxed lognormal clock was detected to be 6.28 ×10^−4^ substitutions per site per year (95% HPD, 5.0×10^−4^ to 7.65 ×10^−4^) under the best fit model. Root of the tree was calculated to be 132 years old [109–157 years 95% HPD, 1882 (1857–1905)]. Time to the most recent common ancestor of the American African genotype and Indian strains of the American African genotype was determined to be 83 years [71–98years 95% HPD, 1931 (1916–1943)]. Lineage India II was estimated to be 58 (95%HPD; 53–65) years old, India I as 19 (95%HPD; 13–28) years old, India III as 54 (95%HPD; 51–58) years old and Afro India as 70 (95%HPD; 60–84) years old. Bayesian skyline plot of Indian DENV-1 strains showed almost constant population size till the year 1995. Population size decreased slowly between 1995 and 2006 which was followed by a sharp decrease between 2006–2011 (Fig 3A).

**Table 2:** Log Marginal Likelihoods by Path Sampling and Stepping Stone sampling for DENV-1 & DENV-3.

	DENV-1		DENV-3	
Model	Log Marginal Likelihood (using Path Sampling)	Log Marginal Likelihood (using Stepping Stone Sampling)	Log Marginal Likelihood (using Path Sampling)	Log Marginal Likelihood (using Stepping Stone Sampling)
Strict Clock, Constant Population	-2687.11	-2687.53	-2252.46	-2253.54
Strict clock, Skyline	-2684.10	-2684.70	-2239.11	-2240.04
Uncorrelated Relaxed Lognormal Clock, Constant Population	-2371.82	-2372.96	-2277.88	-2278.68
Uncorrelated Relaxed Lognormal Clock, Skyline	-2364.56	-2365.91	-2226.80	-2228.36
Uncorrelated Relaxed Exponential Clock, Constant Population	-2375.09	-2375.62	-2244.73	-2246.31
Uncorrelated Relaxed Exponential Clock, Skyline	-2371.63	-2372.96	-2230.11	-2231.01

### Phylogenetic analysis of DENV-3 strains

**Fig 4:**
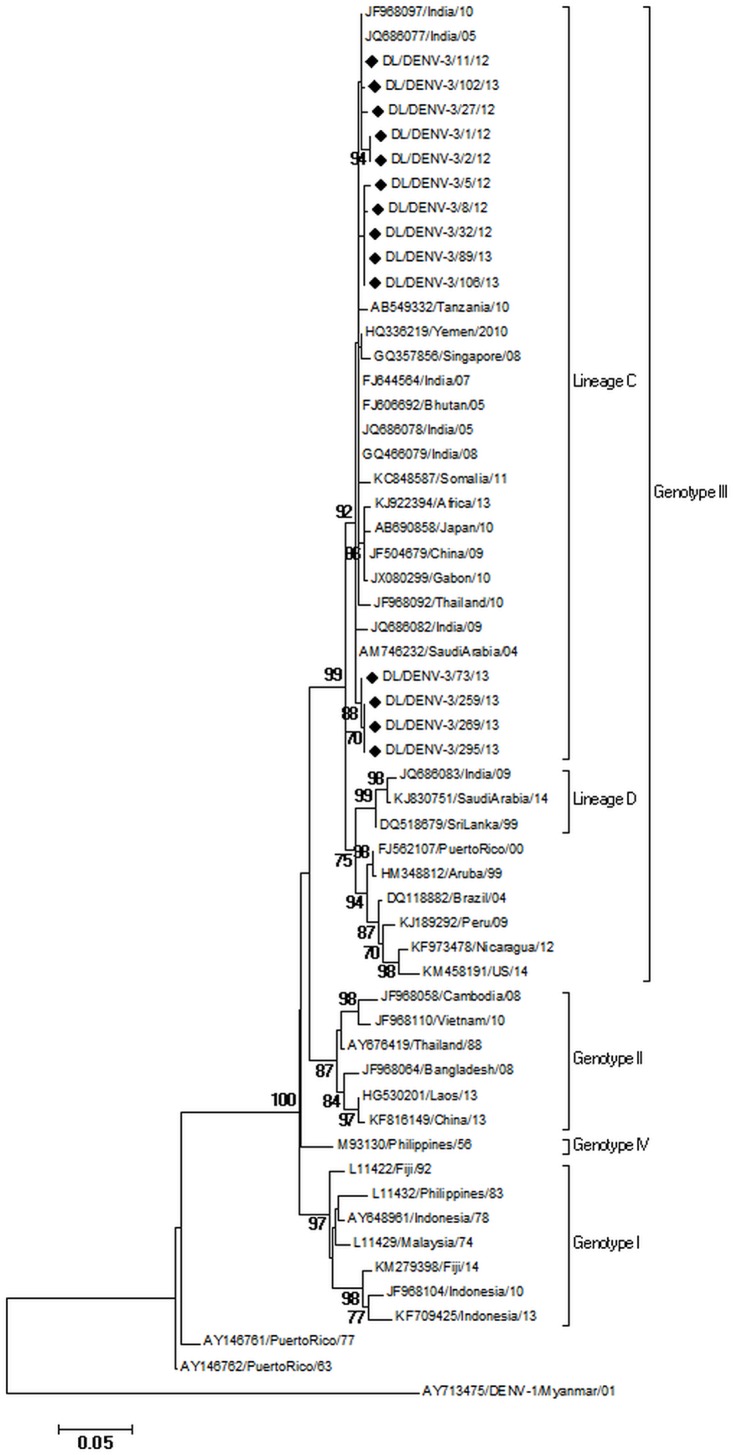
Maximum Likelihood Phylogenetic tree of DENV-3 strains. Strains sequenced in the study are marked by diamonds. Numbers on nodes indicate bootstrap support generated by 1000 replicates. Bootstrap values of >70 are shown.

Fourteen DENV-3 strains were sequenced for the Envelope gene. These were deposited in Genbank with accession numbers KJ729173-KJ729179 and KT026312- KT026318. Phylogenetic analysis of the sequences with accession number KJ729173-KJ729179 has been described in our previous publication [7]. A 504 bp (168 amino acid) region of E gene of 47 DENV-3 strains spanning 1607 to 2110 bp of the DENV-3 genome and 225 to 392 amino acid of the E protein (numbering based on the prototype H87 strain; GenBank Accession Number: M93130) was aligned. The DENV- 3 strains of the present study grouped within Lineage C (as identified by Patil et al. [4]) of genotype III (Fig 4). DENV-3 strains of the study showed nucleotide distances of up to 1.6% while amino acid sequences were identical. Nucleotide distance of 5.6 to 6.5% and amino acid distance of 3.6% were detected in comparison to the prototype H87 strain. The 2012 DENV-3 strains showed nucleotide distances of upto 1.2%. The strains of 2013 also showed genetic distances up to 1.2%. Nucleotide distances between 2012 and 2013 strains were upto 1.6%. Six mutations, all previously reported [19] were detected in the sequenced strains; LysE225Glu, ThrE270Asn, LysE291Glu, LeuE301Thr, LysE383Asn, Arg391Lys with reference to the prototype.

### Bayesian MCMC analysis of DENV-3 strains

**Fig 5:**
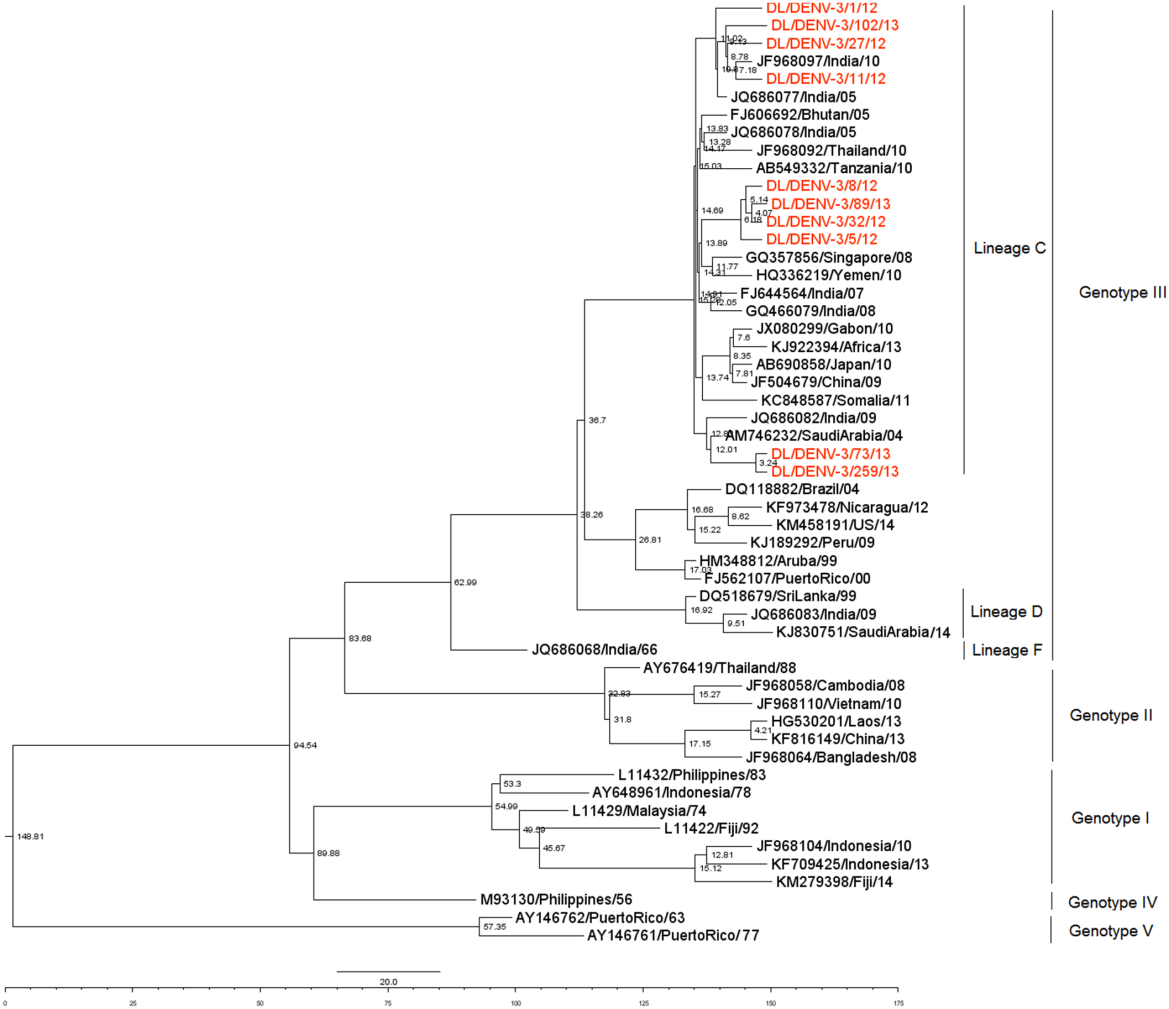
Maximum Clade Credibility tree of Dengue-3 viruses. Tree derived with the best fit model. Node ages are shown at each node.

Bayesian evolutionary analysis for DENV-3 was conducted using general time reversible model of nucleotide substitution with gamma distributed rate variation among sites (4 categories) and proportion of invariant sites (GTR+G+I as selected by MODELTEST 3.7). Uncorrelated relaxed lognormal clock and Bayesian skyline tree prior was chosen as the best fit model as it was favoured by Bayes factor (Table 2). Maximum Clade Credibility Tree was constructed with this model as shown in Fig 5. The mean nucleotide substitution rate under the relaxed lognormal clock was detected to be 7.64 ×10^−4^ substitutions per site per year (5.5×10^−4^ to 9.9 ×10^−4^) under the best fit model. Root of the tree was calculated to be 149 years old [99–201years 95% HPD, 1865 (1813–1915)]. Time to the most recent common ancestor of Genotype III and Indian strains of genotype III was determined to be 63 years [53–74 years 95% HPD, 1951(1940–1961)]. Lineage C was shown to have age of 15 years [11–19 years 95% HPD, 1999 (1995–2003]. Coalescent based reconstruction of demographic history as depicted in the Bayesian skyline plot showed that DENV-3 population size in India increased around 2005 and remained constant till 2013 (Fig 3B).

## Discussion

The present study reports the prevalence of dengue viruses in the city of New Delhi, India. Phylogenetic analysis of the sequenced DENV-1 and 3 strains is also reported. We also conducted molecular clock analysis by Bayesian methods. Further we have also constructed Bayesian skyline plots for Indian DENV-1 and 3 strains. We detected dengue virus RNA in two third of the tested samples by RT-PCR. Dengue serotype 2 was detected in 80% of the dengue positive samples while dengue serotype 4 was not detected at all. Dengue 1 was detected in 26% and dengue 3 in 11% of the samples. The phylogenetic and molecular clock analysis of Dengue 2 viruses is being described in another publication. Our findings are comparable to the serotypic distribution detected in 2009–2012 in Uttar Pradesh, India as reported recently [20]. Dengue 2 was also reported as the major serotype by Das et al. [21] in another study. Similar to our results, dengue serotype 4 was not detected in studies by Mishra et al. and Pandey et al. [20,22].

Dominance of dengue serotype 2 in the present study is a new trend in the epidemiology of dengue in Delhi. A change in serotype dominance has taken place in Delhi. DENV-3 was reported as the dominant serotype from 2003 to 2006 in Delhi [23] while DENV-1 was detected as the predominant serotype in 2008 [24] and 2010 in Delhi [25]. Our serotypic data shows almost equal dominance of Dengue serotype 1 and 2 in the next year (2011) leading to complete dominance of dengue virus type 2 in 2012 and 2013. In 2014, a rise in DENV-1 infections was again detected. Concurrent infections were detected in 18% of the dengue positive cases revealing a high proportion of such cases in Delhi. Detection of co-infection cases is an advantage of RT-PCR based studies as such cases cannot be detected by serological tests. Co-infection by dengue serotypes has been previously reported from India [26,27].

In the present study we conducted sequencing and phylogenetic analysis of DENV-1 and DENV-3 strains circulating in Delhi, India. Phylogenetic analysis of DENV-1 strains clustered the newly sequenced strains with the previously circulating lineage India II [17] of American African genotype. Four lineages of DENV-1 have been reported to have circulated in India. India III and Afro- India have not been detected in recent years. Lineage India II is an old lineage (TMRCA 58 years) which has been detected in India since 1962. Apart from Patil et al., four lineages of a single genotype of DENV-1 (genotype III) in India has been also reported by Kukreti et al. [3] based upon CprM sequences. Apart from the American African genotype, Asian genotype has been also reported from India in 1997 and 1998 [3] but this genotype has not been detected since that study. Nucleotide substitution rate of DENV-1 viruses was determined in the study which is similar to the earlier reported rate of 6.5×10^−4^ substitutions/site/year reported by Patil et al [17]. The TMRCA of American African genotype and Indian strains of this genotype coincide. This estimate is strengthened by a previous report of India being predicted the ancestral state of American African genotype [28].

“The demographic history of a population leaves a signature in the genomes of its modern representatives” [29]. Bayesian skyline plots for Indian DENV-1 strains were constructed in the study. The plot shows changes in the median estimate of relative genetic diversity (Ne τ) of the virus with time where Neτ is the product of effective population size (Ne) and generation time (τ). The plot also shows 95% highest probability density intervals which represents both phylogenetic and coalescent uncertainty. We detected a decrease in population size in Indian DENV-1 strains since 2006 which is in compliance to its low level of detection in recent years.

DENV- 3 strains of the present study grouped within Lineage C of genotype III which is in continuous circulation in India since 2005 [4]. The mean nucleotide substitution rate under the relaxed lognormal clock is similar to the previously reported rate of 8.9 ×10^−4^ [30]. Root of the tree and TMRCA of Genotype III are comparable to that reported by Araujo et al. [30]. TMRCA of lineage C is similar to Patil et al.[4]. Bayesian skyline plot of DENV-3 showed increase in population size around 2005 which is also supported by a DENV-3 outbreak in 2006 [31] in India.

In conclusion, we reported a change in circulating serotype in Delhi, India in recent years. Phylogenetic and molecular dating analysis of DENV-1 and DENV-3 was also carried out in the study. Further Bayesian skyline plots of Indian DENV-1 & 3 strains were also reported. The present investigation will assist in designing control strategies for the epidemics. Further these molecular epidemiological studies will also help us to determine the evolutionary pattern of this emerging virus.
